# Childhood trauma subtypes and specific symptom expression in first-episode psychosis

**DOI:** 10.1192/j.eurpsy.2026.10524

**Published:** 2026-07-31

**Authors:** S. Hamzaoui, K. Mahfoudh, S. Walha, R. Lansari, A. Larnaout

**Affiliations:** Department of psychiatry D, Razi Hospital, Manouba, Tunisia

## Abstract

**Introduction:**

Childhood trauma is a well-established risk factor for psychosis. Beyond its global effect on symptom severity and prognosis, emerging evidence suggests that distinct trauma subtypes may be linked to specific psychotic symptoms.

**Objectives:**

This study examined the associations between childhood maltreatment and individual symptom dimensions in first-episode psychosis (FEP).

**Methods:**

We recruited 65 outpatients with FEP stabilized for at least three months. Childhood trauma was assessed with the Childhood Trauma Questionnaire–Short Form (CTQ-SF), and symptoms with the Positive and Negative Syndrome Scale (PANSS). Correlations were explored between CTQ subscales and PANSS items. A p-value < 0.05 was considered statistically significant.

**Results:**

The total CTQ score correlated positively with delusions (p=0.017, r=0.307), suspiciousness/persecution (p=0.025, r=0.289), unusual thought content (p=0.002, r=0.394), poor judgment and lack of insight (p=0.049, r=0.256), and poor impulse control (p=0.033, r=0.276). The significant correlations between CTQ subscales ans PANSS items are summarized in Table 1.

**Table 1.** Correlations between types of Childhood adversity and PANSS Items Table 1. Long description.

Type of Adversity	PANSS Items	p-value	r
Emotional abuse	Delusions	**0.004**	**0.369**
	Suspiciousness/Persecution	**0.009**	**0.335**
	Unusual thought content	**0.001**	**0.425**
Physical abuse	Delusions	**0.009**	**0.334**
	Suspiciousness/Persecution	**0.042**	**0.263**
	Unusual thought content	**0.000**	**0.440**
Sexual abuse	Poor judgment and lack of insight	**0.036**	**0.271**
Emotional neglect	Inattention	**0.025**	**0.290**
Physical neglect	Inattention	**0.008**	**0.341**

**Conclusions:**

Our findings highlight the impact of early adversity on psychosis expression. Abuse was strongly related to positive symptoms while neglect was linked to attentional and social deficits. These results support the importance of trauma-based, individualized approaches in early intervention.

**Disclosure of Interest:**

None Declared

## Long descriptions

### Table 1. Long description

The table consists of four columns: Type of Adversity, P A N S S Items, p-value, and r.

* Emotional abuse correlates with three items: Delusions (p = 0.004, r = 0.369), Suspiciousness/Persecution (p = 0.009, r = 0.335), and Unusual thought content (p = 0.001, r = 0.425).

* Physical abuse correlates with three items: Delusions (p = 0.009, r = 0.334), Suspiciousness/Persecution (p = 0.042, r = 0.263), and Unusual thought content (p = 0.000, r = 0.440).

* Sexual abuse correlates with Poor judgment and lack of insight (p = 0.036, r = 0.271).

* Emotional neglect correlates with Inattention (p = 0.025, r = 0.290).

* Physical neglect correlates with Inattention (p = 0.008, r = 0.341).

All listed p-values and r-values are presented in bold text.

Navigate back to Table 1..

